# Cost-effectiveness of screening strategies to detect heart failure in patients with type 2 diabetes

**DOI:** 10.1186/s12933-016-0363-z

**Published:** 2016-03-22

**Authors:** Anoukh van Giessen, Leandra J. M. Boonman-de Winter, Frans H. Rutten, Maarten J. Cramer, Marcel J. Landman, Anho H. Liem, Arno W. Hoes, Hendrik Koffijberg

**Affiliations:** Julius Center for Health Sciences and Primary Care, University Medical Center Utrecht, Utrecht, The Netherlands; Department of Clinical Epidemiology and Medical Technology Assessment, University Medical Center Maastricht, Maastricht, The Netherlands; Department of Scientific and Contract Research, Center for Diagnostic Support in Primary Care (SHL-Groep), Etten-Leur, The Netherlands; Amphia Academy, Amphia Hospital, Breda, The Netherlands; Department of Cardiology, Division Heart and Lungs, University Medical Center Utrecht, Utrecht, The Netherlands; Cardiologist, Leusden, The Netherlands; Department of Cardiology, Sint Franciscus Hospital, Rotterdam, The Netherlands; Department of Health Technology and Services Research, MIRA Institute for Biomedical Technology and Technical Medicine, University of Twente, Enschede, The Netherlands

**Keywords:** Heart failure, Type 2 diabetes, Screening, Quality of life, Cost-effectiveness

## Abstract

**Background:**

Heart failure (HF), especially with preserved ejection fraction (HFpEF) is common in older patients with type 2 diabetes (T2DM), but often not recognized. Early HF detection in older T2DM patients may be worthwhile because treatment may be initiated in an early stage, with clear beneficial treatment in those with reduced ejection fraction (HFrEF), but without clear prognostic beneficial treatment in those with HFpEF. Because both types of HF may be uncovered in older T2DM, screening may improve health outcomes at acceptable costs. We assessed the cost-effectiveness of five screening strategies in patients with T2DM aged 60 years or over.

**Methods:**

We built a Markov model with a lifetime horizon based on the prognostic results from our screening study of 581 patients with T2DM, extended with evidence from literature. Cost-effectiveness was calculated from a Dutch healthcare perspective as additional costs (Euros) per additional quality-adjusted life-year (QALY) gained. We performed probabilistic sensitivity analysis to assess robustness of these outcomes. Scenario analyses were performed to assess the influence of the availability of effective treatment of heart failure with preserved ejection fraction.

**Results:**

For willingness to pay values in the range of €6050/QALY–€31,000/QALY for men and €6300/QALY–€42,000/QALY for women, screening-based checking the electronic medical record for patient characteristics and medical history plus the assessment of symptoms had the highest probability of being cost-effective. For higher willingness-to-pay values, direct echocardiography was the preferred screening strategy. Cost-effectiveness of all screening strategies improved with the increase in effectiveness of treatment for HFpEF.

**Conclusions:**

Screening for HF in older community-dwelling patients with T2DM is cost-effective at the commonly used willingness-to-pay threshold of €20.000/QALY by checking the electronic medical record for patient characteristics and medical history plus the assessment of symptoms. The simplicity of such a strategy makes it feasible for implementation in existing primary care diabetes management programs.

## Background

Type 2 diabetes mellitus (T2DM) is known to increase the risk for cardiovascular disease, including coronary artery disease and ‘diabetic cardiomyopathy’ [1, 2]. Heart disease is hence a major cause of death among T2DM patients [1, 3–5]. In a screening study, we recently showed that among T2DM patients aged 60 years or over, 27.7 % had unrecognized heart failure (HF); 22.9 % HF with preserved ejection fraction (HFpEF) and 4.8 % HF with reduced ejection fraction (HFrEF) [5]. For HFrEF prognostically beneficial treatment is available, while this is lacking for HFpEF [6]. New drugs for HFpEF, however, are currently under investigation.

Early HF detection in older T2DM patients may be worthwhile because treatment may be initiated in an early stage. Moreover, adequate diagnosis of HF may prevent misclassification of symptoms, such as shortness of breath as a respiratory disease [13]. However, in the light of limited healthcare resources, balancing healthcare interventions and costs has become increasingly important. Screening older T2DM patients for HF will induce costs for testing, as well as downstream costs for additional check-ups and treatment. On the other hand, early HF detection may increase health effects through early treatment initiation. As treatment may prevent HF progression, this might save costs on the long-term. If a screening strategy shows it can achieve health effects at acceptable costs, large-scale implementation within existing primary care diabetes management programs may be advocated. Currently, there is no such HF screening program among older community-dwelling T2DM patients diabetes in the Netherlands. We therefore assessed the long-term health effect and costs of five screening strategies to detect HF in T2DM patients aged 60 years or over in the Dutch primary care setting.

## Methods

### The UHFO-DM2 study

We conducted the ‘UHFO-DM2’ screening study from February 2009 to March 2010, within which participants were followed for 1 year [7]. All T2DM patients enlisted in primary care practices in the South-West of the Netherlands were eligible to participate. In total 581 patients who did not already have a cardiologist-confirmed diagnosis of HF and gave written informed consent were included in the study. The study complied with the declaration of Helsinki and was approved by the institutional review and ethics boards of the University Medical Center Utrecht and the Admiraal de Ruyter Hospital in Goes, the Netherlands. The study is registered in the register of the Central Committee on Research Involving Human Subjects (NL22717.041.08).

All 581 participants underwent a 1-day standardized diagnostic assessment including history taking, physical examination, electrocardiography (ECG), blood tests, and echocardiography. Presence of HF (HFpEF or HFrEF) was determined by an expert-panel consisting of two cardiologists and an experienced general practitioner (GP) who followed the criteria for HF of the recent European Society of Cardiology guidelines [2, 7]. Preference-based utilities were measured with the EuroQol-5D (EQ5D) instrument during the baseline visit [8]. After 3 and 12 months the participants filled out the same questionnaires, plus an additional questionnaire on medication use.

### Overview of the screening strategies

We assessed the cost-effectiveness of five screening strategies:

1. *EMR/symptoms*; a strategy based on information available from the Electronic Medical Record (EMR) of general practitioners, i.e., age, gender, history of ischaemic heart disease, asthma or COPD, hypertension, peripheral arterial disease, transient ischaemic attack or stroke, combined with the assessment of presence of the following symptoms; dyspnea, fatigue, ankle oedema, nocturia, or palpitations.
2. *EMR/symptoms/PhysicalExam*; strategy 1 plus features from the physical examination (PE): pulmonary crepitations, jugular venous distension, ankle oedema, or a laterally displaced apex beat.
3. *EMR/symptoms/PhysicalExam/NTproBNP*; strategy 2 plus measurement of natriuretic peptide (NTproBNP) applying the exclusionary cut-point of 15 pmol/L (≈125 pg/ml).
4. *EMR/symptoms/PhysicalExam/NTproBNP/ECG*; strategy 3 plus ECG.
5. *Echocardiography*; everybody is directly referred for echocardiography.

Strategy 1 to 4 are based on recently developed diagnostic screening models to estimate the risk of HF in T2DM patients [9]. A risk of <20 % was used for ruling out HF, and to estimate the sensitivity and specificity of the different strategies [10]. In our screening models, patients were referred for echocardiography if the HF risk was ≥20 %. All the initial, once-in-a-life screening strategies were followed by annual assessment of *EMR/symptoms*.

### Model structure

**Fig. 1:**
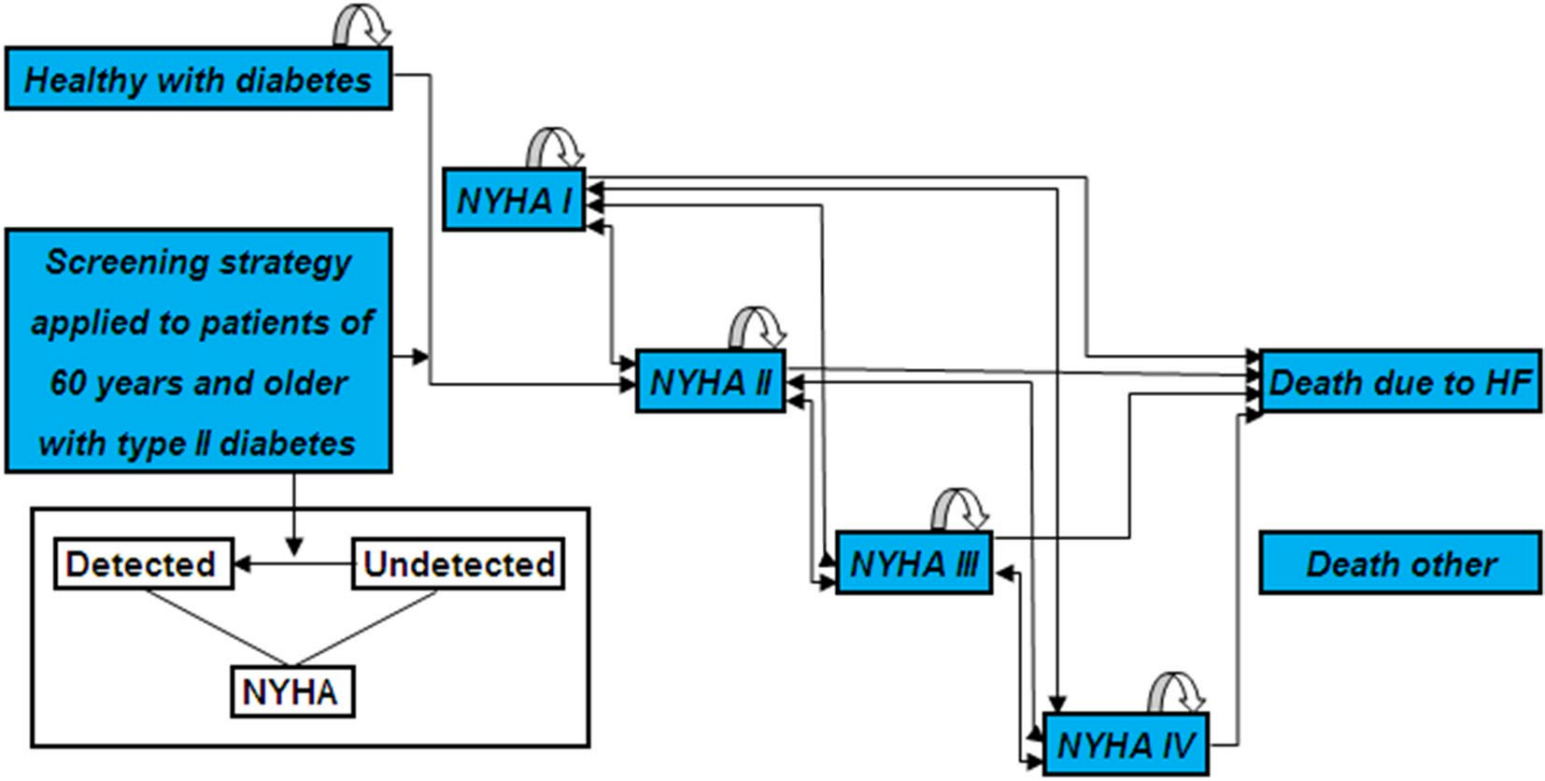
Schematic representation of the model structure. *NYHA* New York Heart Association, *HF* heart failure. Within each NYHA state patients can either have detected or undetected heart failure. Following the screening strategy patients can be diagnosed with heart failure and transition from the undetected to the detected state may take place. Patients can transition from NYHA IV to NYHA I because of the (small) probability of transitioning to a better NYHA state in 1 month [22]. From the diabetes without HF and any of the NYHA states individuals can die from causes other than HF and transition to ‘Death other’

To assess long-term consequences in terms of costs and health effects of the five screening strategies compared to no screening (usual care), a Markov decision analytic cohort model of HF progression was developed (Fig. 1). Markov models assume that a patient is always in one of the predefined health states. In this, here representing HF progression, and use transition probabilities to allow patients to transition between health states over time. Each health state is assigned a (time-dependent) utility and cost. The long-term expected health effects and costs of each (screening) strategy can be calculated by multiplying the total time spent in each state by the utilities and costs corresponding to these states, respectively. We refer to the literature for further information. [11–13].

The model was designed to simulate hypothetical cohorts of men and women with T2DM initially screened for HF at age 60. The model employed 3-months’ time cycles, corresponding to the regular interval of diabetes consultations and a lifetime horizon, i.e., the simulation stopped when all 581 patients in the hypothetical cohort had died. In the model, all individuals started in the *diabetes without HF* health state, or one of the four HF states based on the four New York Heart Association (NYHA) classes, subdivided in the categories ‘detected’ or ‘undetected’ [14]. In all screening, strategies individuals suspected of HF underwent echocardiography as the cornerstone investigation, which was assumed to be the reference standard with a sensitivity and specificity of 1.0.

Individuals without HF at the time of the initial screening could develop HF in subsequent years, which would transfer them to the health state *NYHA II undetected*. They could then be detected by the subsequent annual screening similar to the individuals with HF who were missed with the initial screening. Finally, all individuals could die from causes unrelated to HF, while HF patients could also die because of HF.

### Input parameters

The baseline distribution of individuals over the different health states was based on the UHFO-DM2 screening study with a screen-detected prevalence of HF of 27.7 % (22.9 % for HFpEF and 4.8 % for HFrEF) and the sensitivity and specificity of the screening strategy (Table 1) [5]. The risk of death from causes other than HF was based on age- and gender-specific Dutch life tables (Table 2), [15] whereas the risk of death from HF was estimated according to the Seattle Heart Failure (SHF) model [5, 16]. The survival estimates of the SHF model were calculated using the mean predictor values from the UHFO-DM2 cohort, stratified by gender and age categories (60–70, 70–80 and 80+ years). The SHF model parameters *daily defined dose for diuretic use*, *percentage lymphocytes,* and *uric acid* were not obtained in the UHFO-DM2 cohort and therefore derived from the ELITE2 cohort, [3, 17] which was used for the validation of the SHF cohort [5, 16]. The SHF model was developed in cases with HFrEF, whereas in the UHFO-DM2 cohort the majority of newly detected cases of HF had HFpEF (82.6 %) [5, 16]. In the main analysis, we therefore conservatively assumed there is no medication effect for patients with HFpEF [17–19].

**Table 1:** Sensitivity, specificity, and costs of heart failure screening strategies in patients with type 2 diabetes of 60 years or older

Parameters	0 No screening	1 EMR symptoms	2 EMR symptoms Physical exam	3 EMR symptoms Physical exam NTproBNP	4 EMR symptoms Physical exam NTproBNP ECG	5 Echo cardio- graphy	Distribution	Source
Sensitivity^a^								
NYHA 1	0.000	0.250	0.250	0.250	0.500	1.000	Beta	Cohort [5]
NYHA 2	0.000	0.853	0.853	0.879	0.862	1.000	Beta	Cohort [5]
NYHA 3	0.000	0.923	0.949	0.897	0.897	1.000	Beta	Cohort [5]
NYHA 4	0.000	1.000	1.000	1.000	1.000	1.000	Beta	Cohort [5]
Specificity	1.000	0.610	0.617	0.652	0.676	1.000	Beta	Cohort [5]
Screening								
GP	€0.00^b^	€6.39	€15.17	€36.67	€61.77	€0.00^c^	Gamma	Dutch tariff [24–26]
Echocardiography	€169.38	€169.38	€169.38	€169.38	€169.38	€169.38	Gamma	Dutch tariff [24–26]
ECG stress test	€94.75	€94.75	€94.75	€94.75	€94.75	€94.75	Gamma	Dutch tariff [24–26]

For each screening strategy NYHA-specific sensitivities were calculated and for each of these sensitivities a beta-distribution was specified with the true positives and total positives as parameters. Similarly, beta distributions were assigned to the specificities of each of the screening strategies. Gamma distributions with parameters using a variance equal to the mean were used for the costs

*EMR* Electronic Medical Record, *GP* general physician

^a^In general, more extensive screening strategies yielded higher sensitivity and specificity at higher costs, except for NYHA 2 when adding ECG and for NYHA 3 when adding NTproBNP and/or ECG

^b^Strategy costs in case of no screening are kept fixed at €0 in the sensitivity analyses

^c^There are no GP costs here as everyone is, after their regular diabetes checkup, directly sent for echocardiography

**Table 2:** Input parameters for the Markov model men and women with type 2 diabetes of 60 years or older

Parameters	Men		Women		Distribution	Data source
	Detected	Undetected	Detected	Undetected		
Incidence						
(100,000 person-years)	658		666		Fixed	Population estimates 23;24
HF Prevalence						
NYHA I		0.007		0.000	Dirichlet	Cohort study [5]
NYHA II		0.148		0.142	Dirichlet	Cohort study [5]
NYHA III		0.047		0.031	Dirichlet	Cohort study [5]
NYHA IV		0.000		0.000	Dirichlet	Cohort study [5]
Mortality (year)	0.010		0.007		Fixed	Population estimates [20]
HF Mortality (year)						
NYHA I	0.042	0.043	0.035	0.036	Fixed	Cohort estimate [5]
NYHA II	0.066	0.067	0.056	0.057	Fixed	Cohort estimate [5]
NYHA III	0.103	0.105	0.087	0.089	Fixed	Cohort estimate [5]
NYHA IV	0.159	0.163	0.137	0.139	Fixed	Cohort estimate [5]
Annual HF costs^a^						
NYHA I	€1777	€1786	€1172	€1100	Gamma	Cost study [38]
NYHA II	€2099	€2114	€1370	€1302	Gamma	Cost study [38]
NYHA III	€3235	€3275	€2070	€2018	Gamma	Cost study [38]
NYHA IV	€8752	€8912	€5470	€5490	Gamma	Cost study [38]
Medication prescription						
ACE-inhibitors	0.53	0.53	0.27	0.18		Cohort study [5]
Beta-blockers	0.57	0.50	0.50	0.50		Cohort study [5]
Utilities^b^						
Diabetes without diagnosed HF	0.868				Beta	Cohort study [5]
NYHA I	0.855	0.817			Beta	Cohort study [5]
NYHA II	0.790	0.739			Beta	Cohort study [5]
NYHA III	0.734	0.685			Beta	Cohort study [5]
NYHA IV	0.665	0.683			Beta	Cohort study [5]

^a^Annual HF costs were mostly higher for undetected than for detected HF patients. Annual HF costs for detected female HF patients in NYHA class I-III were somewhat higher than for undetected female HF patients in corresponding NYHA classes because of higher use of primary care and medication

^b^Utilities were assumed to be the same in men and women. Utilities were higher for detected than for undetected HF patients except for NYHA class IV, where utilities were 0.665 and 0.683, respectively

Medication prescription of patients with undetected HF was based on the observed proportion of individuals in the UHFO-DM2 cohort using specific medication prior to their HF diagnosis. For detected HF the observed proportion of medication use 1 year after diagnosis in the UHFO-DM2 cohort was used (Table 2). With modeling, compliance to prescribed medication was assumed as no evidence for this patient group was available. The probabilities of healthy individuals to develop HF were based on age- and gender- stratified HF incidence in the Netherlands, taking into account the presence of diabetes [20, 21]. Transition probabilities between different NYHA-states were derived from 1-month transition probabilities of 65-years old HF patients who are on medical therapy [22]. With increasing age, the probability to regress to a worse state (higher NYHA-class) was increased similar to the increasing mortality risk according to the SHF model in the corresponding NYHA classes, taking into account whether the patient had detected or undetected HF [16]. The probability to regress to a better state was calculated accordingly.

### Health outcomes

Health effects were determined by calculating expected life-years and quality-adjusted life-years (QALYs). Utilities for each of the eight HF states and the state of *diabetes without HF* were based on the EQ5D-scores obtained in the UHFO-DM2 study (Table 2). The utilities for undetected HF were based on scores obtained before diagnosis, whereas utilities for *diabetes without HF* and detected HF states were based on EQ5D-scores obtained 1 year after diagnosis.

### Costs

Our analysis was conducted from a healthcare perspective, incorporating costs directly related to screening and diagnosis, and HF-related costs for detected and undetected HF patients within different NYHA-classes. Costs for screening and diagnosis included costs for investigations and consultation and, in case of a calculated risk of HF > 20 %, a cardiologist consultation including echocardiography. If HF was diagnosed, costs of a cardiac ECG stress test were added. In the Netherlands, it is common practice for cardiologists to perform this test as a first step in the assessment if angina pectoris or myocardial ischaemia is involved [23]. All costs were based on tariffs set by the Dutch Healthcare Authority (1 January 2011) according to Dutch guidelines (Table 1) [24–26].

Long-term HF-related costs were split up into costs for all HF patients (hospitalization and nursing care) and costs for patients with *detected* HF (extra GP consultation and medication use), stratified by gender and age. All T2DM patients have regular GP consultations and receive anti-diabetes medication and the vast majority receives blood pressure lowering drugs and statins. We therefore solely used the *increase* in costs related to the detection of HF. Costs for all HF patients were additionally weighted over NYHA classes using hazard ratios for hospitalization per NYHA class, which were adjusted for detected and undetected HF using the ratio of their mortality risks based on the SHF model [16, 22].

### Analyses

Life-years, QALYs, and costs per person were calculated over a lifetime horizon, with costs and health effects discounted at 4 and 1.5 % per year, respectively, according to Dutch guidelines [27]. To assess robustness of the results given the uncertainty in the evidence (Table 2), probabilistic sensitivity analyses were performed using Monte Carlo simulation based on 10,000 samples. The incremental cost-effectiveness ratios (ICERs) were calculated by first ranking all strategies in order of improving health outcomes and calculating the additional costs of obtaining these health outcomes [12]. Strategies offering no additional health benefits, or additional health benefits at too high costs compared to other strategies were considered ‘dominated’, i.e., less favourable and not cost-effective, which was visualized in a cost-effectiveness frontier. Model development and analyses were done with Microsoft Excel 2010.

### Scenario analyses for HFpEF medication

We distinguished between screening for HFrEF and HFpEF, because the prevalence is much higher for HFpEF, while convincing evidence-based therapy is only available for HFrEF. Treatment of HFpEF is focused on reduction of symptoms of fluid overload, adequate blood pressure control and management of comorbid conditions [2]. Diuretics are the only option for symptom relief, but their prognostic effects have never been adequately evaluated. Other drugs, including beta-blockers, angiotensin-converting-enzyme inhibitors, angiotensin II receptor blockers and mineralo-corticoid inhibitors have been tested in randomized-controlled trials in patients with HFpEF, mainly in addition to diuretics, with at the best a statistically non-significant relative risk reduction in all-cause mortality of around 10 % [2].

Currently, medication for HFpEF has only shown at best a tendency to a mortality-reducing effect. [17, 28–31] Therefore, scenario analyses were performed to assess the potential cost-effectiveness if evidence on effective medication for HFpEF becomes available in the future. We varied the effectiveness of medication for patients with HFpEF from 0 to 100 %, with increments of 10 %, of the effectiveness for HFrEF. Hence, 100 % implies equal effectiveness in HFpEF as in HFrEF. Incremental QALYs and costs of the screening strategies were evaluated for each of these HFpEF medication effectiveness scenarios. [17, 28–31] Scenario analyses were performed to assess the potential cost-effectiveness if mortality reducing effective medication for HFpEF becomes available in the future. We varied the effectiveness of medication for patients with HFpEF from 0 to 100 % effectiveness compared to the effect in HFrEF, with increments of 10 %, i.e., 100 % implies equal effectiveness in HFpEF as in HFrEF. The ICER of the screening strategies was evaluated for each of these scenarios of medication effectiveness in HFpEF.

## Results

**Fig. 2:**
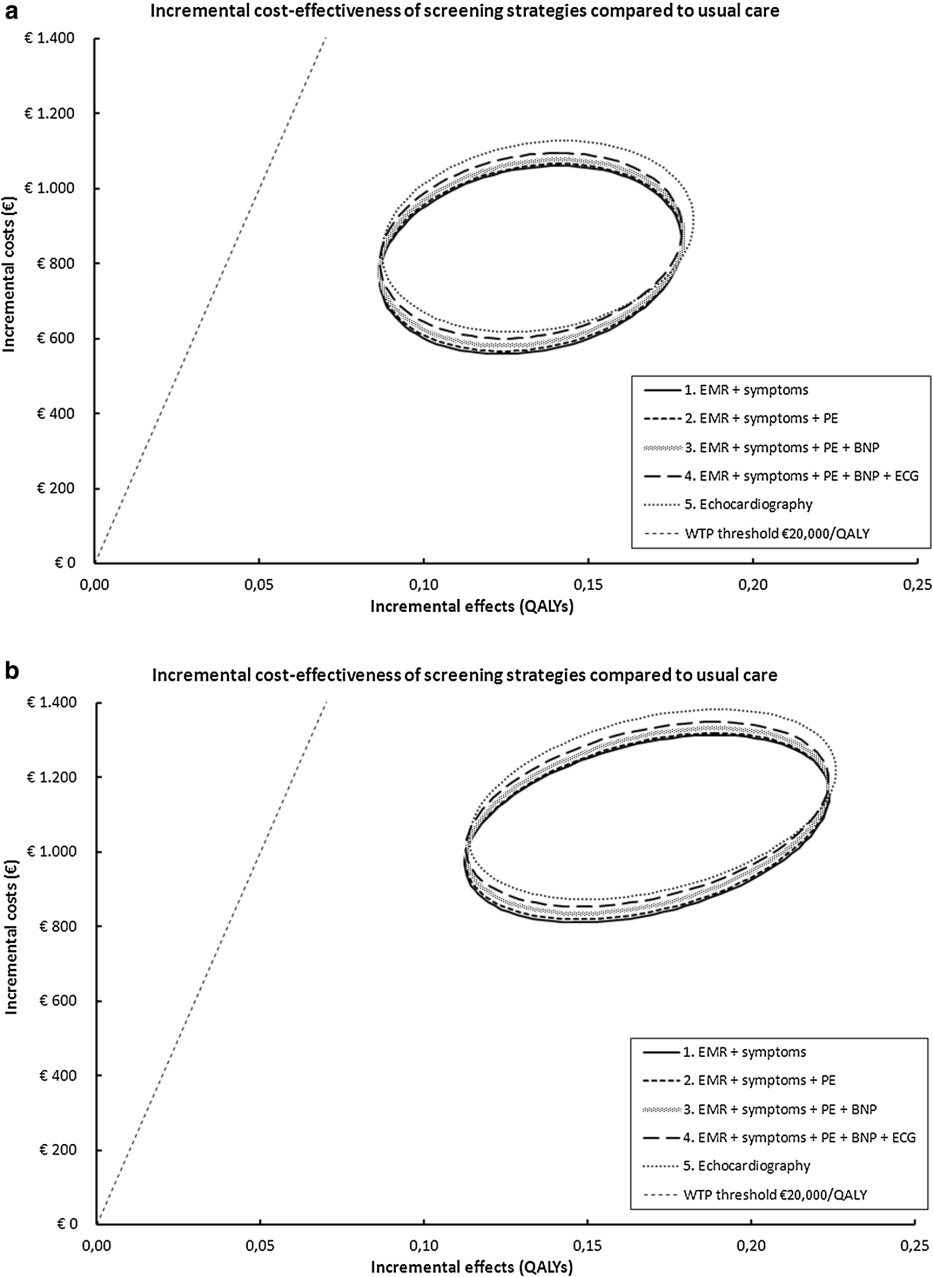
Incremental cost-effectiveness planes for patients with type 2 diabetes of 60 years or older. **a** Men. **b** Women

The average expected life-years and QALYs for 60-year old men and women with T2DM increased with HF screening, starting at the age of 60. For men, the expected life-years were 14.726 (12.345 QALYs) for *usual care* (no screening) and 14.742 (12.477 QALYs) if *EMR/symptoms* or strategy 2–4 were used. If echocardiography was performed straightaway in all patients (strategy 5), expected life-years were 14.742 and QALYs 12.479. For women, expected life-years increased from 16.830 (14.047 QALYs) with *usual care* to 16.851 (14.215 QALYs) if the *EMR/symptoms* strategy was implemented, and to 16.852 (14.217 QALYs) with the *Echocardiography* strategy. For *usual care*, expected lifetime costs for men and women were €6795 and €5024, respectively. These costs increased if screening strategies were performed, with the highest costs for the *Echocardiography* strategy; €7667 for men and €6152 for women (Table 3). The incremental cost-effectiveness planes for the five screening strategies as compared to *usual care* (no screening) are presented in Fig. 2.

**Table 3:** The incremental comparison of expected life years, QALYs, costs, and ICERs for five screening strategies for heart failure in patients with type 2 diabetes of 60 years or older

Strategy	0 No screening	1 EMR symptoms	2 EMR symptoms Physical Exam	3 EMR symptoms Physical exam NTproBNP	4 EMR symptoms Physical exam NTproBNP ECG	5 Echocardiography
Life expectancy (years)						
Men	14.726	14.742	14.742	14.742	14.742	14.742
Women	16.830	16.851	16.851	16.851	16.851	16.852
QALY expectancy (years)						
Men	12.345	12.477	12.477	12.477	12.477	12.479
Women	14.047	14.215	14.215	14.216	14.215	14.217
Expected costs pp (euros)						
Men	€6795	€7605	€7611	€7625	€7642	€7667
Women	€5024	€6086	€6093	€6107	€6125	€6152
Strategy comparison	NA	1 vs 0	2 vs 1	3 vs 1	4 vs 1	5 vs 1
Additional QALYs to comparator						
Men	NA	0.132	0.000	0.000	0.000	0.002
Women	NA	0.168	0.000	0.000	0.000	0.002
Additional costs to comparator						
Men	NA	€810	NA	NA	NA	€62
Women	NA	€1063	NA	NA	NA	€66
ICER						
Men	NA	€6115^a^	Dominated	Dominated	Dominated	€29,100
Women	NA	€6318^a^	Dominated	Dominated	Dominated	€39,326

In the upper part of the table the absolute expected life-years, QALYs, and costs are given for each strategy. In the lower part, for each strategy these life-years, QALYs and costs are compared to the current optimal strategy

*EMR* Electronic Medical Record, *ICER* incremental cost-effectiveness ratio

^a^This is the strategy expected to be optimal for a WTP of €20,000 per QALY

**Fig. 3:**
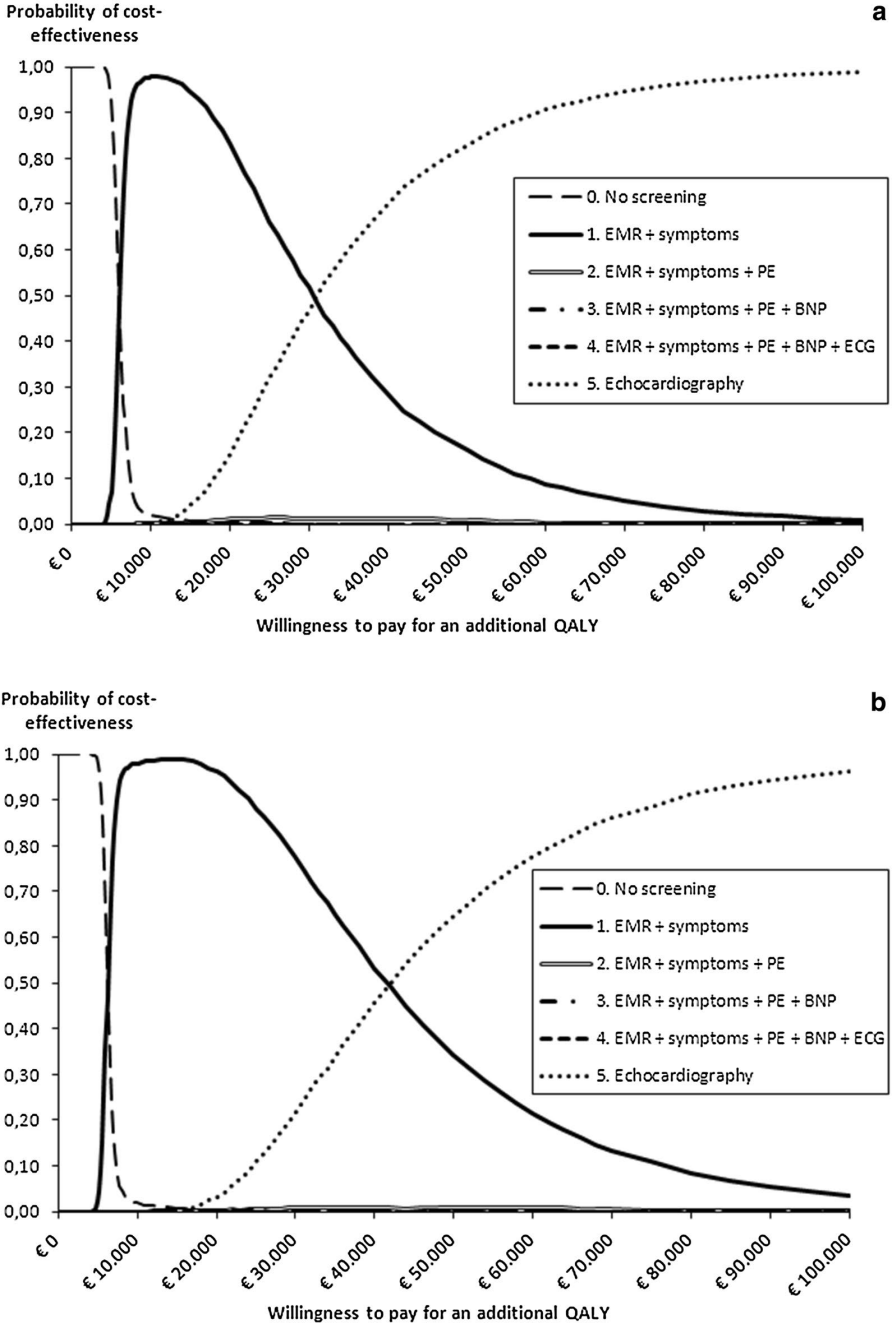
Cost-effectiveness acceptability curves for patients with type 2 diabetes of 60 years or older. **a** Men. **b** Women

**Fig. 4:**
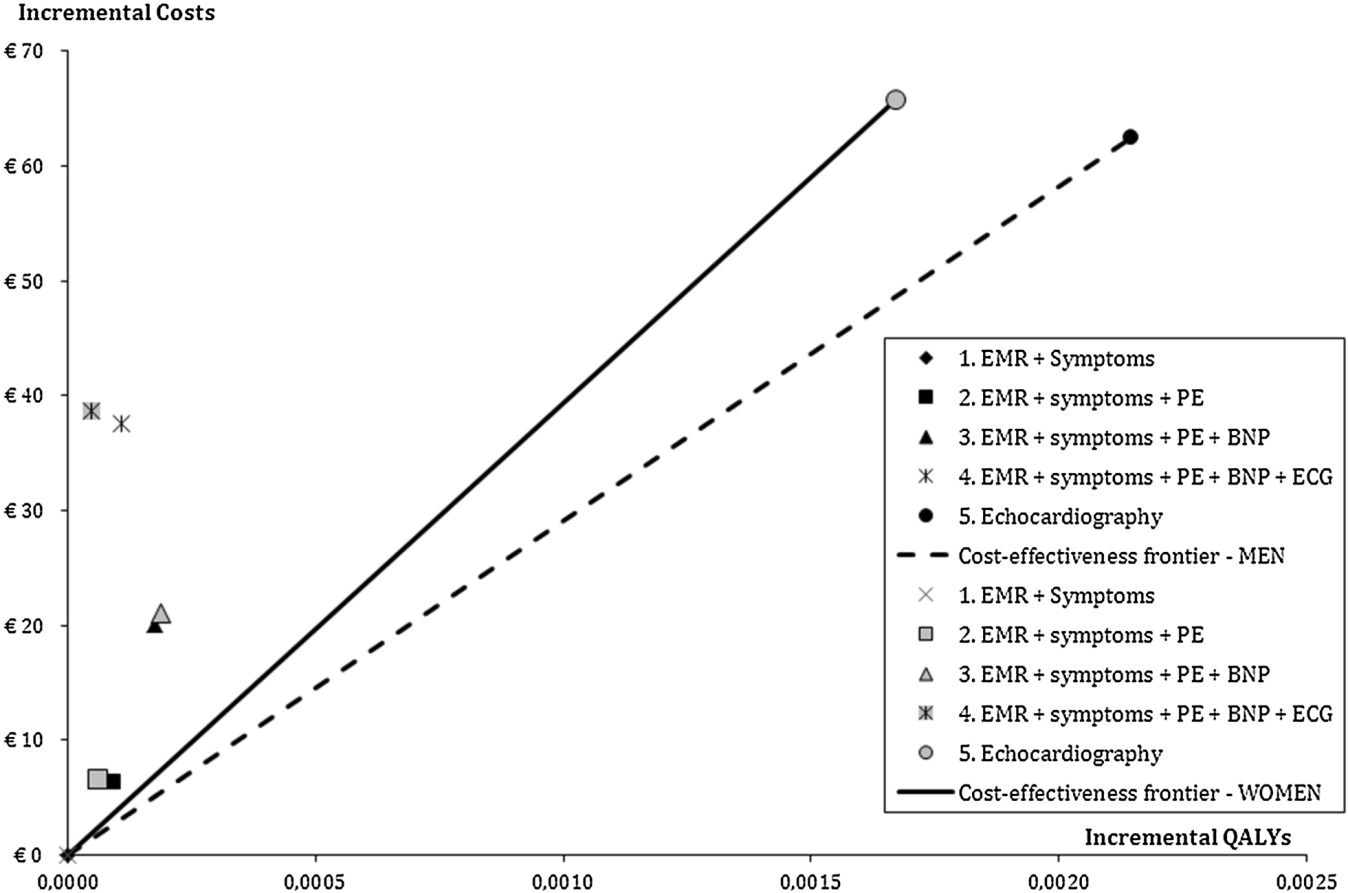
Cost-effectiveness frontier patients with type 2 diabetes of 60 years or older. For each strategy life-years, QALYs and costs are compared to the current optimal strategy (lower part Table 3). The results are visualized in this cost-effectiveness frontier, which uses strategy 1 (EMR + symptoms) as the reference point, i.e., the origin

The cost-effectiveness acceptability curves (Fig. 3) showed that the *EMR/symptoms* strategy is the preferred strategy for a willingness to pay (WTP) ranging from €6050/QALY to €31,000/QALY for men and €6300/QALY to €42,000/QALY for women. For lower WTP-values, the *usual care* (no screening) strategy was optimal, while for higher WTP-values the *Echocardiography* strategy is the preferred strategy. Comparing the different screening strategies and evaluating ICERs showed that QALYs could only be gained on top of the *EMR/symptoms* strategy by the *Echocardiography* strategy, and not by the other three strategies (Table 3 lower part, Fig. 4).

### Scenario analyses for HFpEF medication

The ICERs of all strategies become more favourable with increasing medication effectiveness. For a WTP of €20,000/QALY *EMR/symptoms* is the preferred strategy. Increasing the relative HFpEF medication effectiveness *Echocardiography* is the preferred strategy in men if this relative medication effectiveness is 90 % or more. With a WTP of €80.000 *Echocardiography* is the preferred strategy for any relative effectiveness (Table 4).

**Table 4:** The incremental comparison of ICERs for different medication scenarios

Relative effectiveness	1. EMR + symptoms vs 0. No screening	5. Echocardiography vs 1. EMR + symptoms
Men (%)		
0	€6115	€29,100
10	€5573	€27,552
20	€5067	€26,157
30	€4591	€24,895
40	€4140	€23,749
50	€3709	€22,705
60	€3293	€21,753
70	€2890	€20,880
80	€2494	€20,079
90	€2102	€19,341
100	€1711	€18,660
Women (%)		
0	€6318	€39,326
10	€5888	€38,122
20	€5496	€37,015
30	€5137	€35,998
40	€4807	€35,067
50	€4502	€34,216
60	€4219	€33,442
70	€3995	€32,741
80	€3706	€32,110
90	€3471	€31,547
100	€3248	€31,049

*EMR* Electronic Medical Record, *PE* physical examination *ICER* incremental cost-effectiveness ratio. Relative effectiveness represents HFPEF medication effectiveness as a percentage of the effectiveness in HFREF. For each strategy the incremental life-years, QALYs and costs are compared to the current optimal strategy and the ICER was calculated. Strategies 2, 3, and 4 were dominated by strategy 5

## Discussion

Screening community-dwelling T2DM patients of 60 years or over annually for HF in the Dutch primary care setting results in a small increase in life expectancy and QALYs at relatively low costs. Checking the EMR for risk factors plus assessment of specific symptoms is the preferred initial, once-in-a-life strategy, with an ICER of €6115 for men and €6318 for women as compared to no screening. For a WTP of €20,000/QALY, a commonly used threshold for patient care in Europe, this strategy would thus be cost-effective [26]. If patients with HFrEF would be treated optimally according to the guidelines, instead of e.g., the current uptake of the most beneficial medication in heart failure, betablockers, which was 58 %, the cost-effectiveness of screening T2DM patients will even be more favourable.

In the base-case analysis we assumed no beneficial prognostic effects of treatment for patients with HFpEF. We performed scenario analyses in which we evaluated the cost-effectiveness considering varying prognostic beneficial treatment effects in this type of HF. These scenario analysis showed that if beneficial medication becomes available for HFpEF, screening strategies in older T2DM patients would have a more favourable ICER, reaching to €3709/QALY for men and €4502/QALY for women, assuming a treatment effect for HFpEF that is 50 % of the current treatment effect of HFrEF (Table 4).

### Strengths and limitations of the study

We assessed multiple screening strategies, all feasible to implement in everyday primary care practice. Our model was based on real-life medication prescriptions in screen-detected HF cases from a cohort study in 581 T2DM patients aged 60 years or over [5]. We only applied a single cut-off point for considering HF to be present. However, when evaluating screening strategies often a single cut-off is used and the 20 % cut-off we applied has been used in earlier studies [10].

Although we did account for higher health-care costs for individuals with undetected HF, we did not consider the costs of a possible acute life-threatening exacerbation of HF necessitating hospital admission. Incorporating such a risk in our model would result in much higher costs for the *non*-*screening* strategy, and thus in an even more favorable cost-effectiveness of screening. Furthermore, end-of-life costs and health deterioration in patients dying from HF may be underestimated because in the model patients could die while being in any NYHA state, not considering rapid disease progression. For example, in our model 30 % of the patients would die from HF while being in NYHA class II, and any progression to higher NYHA states with its associated health losses immediately prior to death is not taken into account. However, the majority of HF deaths (64 %) in our model occurred in patients with NYHA class 3 and 4, indicating that this underestimation is limited.

Another limitation of our study is that the SHF model was derived from cohorts with only HFrEF patients, while the majority of patients with HF in the UHFO-DM2 cohort had HFpEF. Moreover, some of the predictor values of the SHF model were not available in the UHFO-DM2 cohort. Therefore, the effectiveness of medication incorporated in the SHF model may not translate perfectly to our cohort. This issue has been addressed by assuming that HFpEF patients do not benefit of any treatment. The additional scenario analyses to assess the impact of the availability of prognostically beneficial medication for HFpEF on ICER were added to further address this issue.

### Implications for research and policy

Our results support screening for HF among T2DM patients of 60 years or over based on the information available from the EMR plus assessment of specific symptoms suggestive of HF, followed by echocardiography in those with a calculated risk of HF of more than 20 %. Even though only for the (small) proportion of patients with HFrEF mortality-reducing medication is available, this screening strategy is cost-effective when using a WTP of €20,000/QALY. Comparing this HF screening to other screening programs, the cost-effectiveness would be better than for cervix screening for instance, which currently costs €20,000-€50,000/QALY [32]. Furthermore, whenever (evidence for) mortality-reducing medication becomes available for HFpEF, of which many experts are convinced [19, 33], the incremental costs/QALY of the screening strategies will decrease considerably, as can be seen in the scenario analyses for effectiveness of HFpEF medication (Table 4). The results of this cost-effectiveness analysis were based on the Dutch setting and may not directly be transferred to other countries. Further research is needed to translate our model to settings in other countries.

Our observations are in line with a registration study, which concluded that despite advances in patient management there is further potential to improve both the detection and management of patients with diabetes and coronary artery disease [34]. Furthermore, relatively tight glucose control has some cardiovascular benefits. HbA1c below 7.0 % as the goal to maximize the cardiovascular benefits remains suspended [35]. In addition, a multidisciplinary risk assessment and management program for patients with diabetes mellitus intervention was associated with lower incidences of individual and total cardiovascular complications, as well as all-cause mortality over 3 years follow-up. This study shows risk assessment was an effective approach to reduce future cardiovascular complications in diabetes patients [36]. Also the ‘St Vincent’s screening to prevent heart failure’ study showed that among patients with cardiovascular risk factors, natriuretic peptide-based screening and collaborative care reduced left ventricular dysfunction, HF and major adverse cardiac events, and has a high probability of being cost-effective [37].

## Conclusions

Screening for heart failure by simply checking the electronic medical record for patient characteristics and medical history plus the assessment of symptoms in community-dwelling patients with type 2 diabetes of 60 years and older is cost-effective at the commonly used willingness-to-pay threshold of €20.000/QALY. The simplicity of such a strategy makes it feasible for implementation in existing primary care diabetes management programs. Cost-effectiveness may improve considerably with the availability of effective treatment for heart failure with preserved ejection fraction.
